# Application of Stationary IoT Sensor Networks to Reduce Exposure to Particulate Matter During Local Hotspot Events in a Megacity: A Fireworks Case Study

**DOI:** 10.3390/s26134307

**Published:** 2026-07-07

**Authors:** Sangwon Bang, Kyung Hwan Kim, Kwang-Rae Kim, Hyun-Ju Ha, Jong-Cheol Yoon, Heejung Jung, Meehye Lee, Seung-Bok Lee

**Affiliations:** 1 Center for Climate and Carbon Cycle Research, Korea Institute of Science and Technology (KIST), Seoul 02792, Republic of Korea; sone971116@naver.com (S.B.);; 2 Graduate School of Energy and Environment (KU-KIST Green School), Korea University, 145 Anam-ro, Seongbuk-gu, Seoul 02841, Republic of Korea; 3 Air Quality Integrated Analysis Center, Seoul Research Institute of Public Health and Environment, Seoul 13818, Republic of Korea; 4 Department of Mechanical Engineering, University of California, Riverside, CA 92521, USA; 5 College of Engineering-Center for Environmental Research and Technology (CE-CERT), University of California, Riverside, CA 92521, USA; 6 Department of Earth and Environmental Sciences, Korea University, 145 Anam-ro, Seongbuk-gu, Seoul 02841, Republic of Korea

**Keywords:** exposure, particulate matter, low-cost air quality sensor network, fireworks, IoT, local hotspot

## Abstract

Growing public concern over the health impacts of particulate matter (PM_2.5_ and PM_10_) has highlighted the need for air quality monitoring networks that can raise an alert when high PM_2.5_ and PM_10_ concentrations are detected. However, existing networks often have insufficient spatiotemporal resolution to detect localized events with high PM_2.5_ and PM_10_ concentrations. In this study, the existing air quality monitoring network in Seoul was complemented by Smart Seoul Data of Things (S-Dot), which is an Internet of Things (IoT)-based sensor network that collects various types of urban data. S-Dot sensors were used to track the spatial distribution and temporal evolution of high-concentration plumes generated by a fireworks festival, and the PM_2.5_ and PM_10_ concentrations measured by the S-Dot sensors were corrected with data measured by precision instruments at the closest air quality monitoring station. While the existing high-concentration alert system is designed to issue warnings when averaged PM_2.5_ concentrations measured at twenty-five air quality monitoring stations across the entire urban area exceed a threshold for a specified duration, the high spatial resolution of S-Dot was leveraged to provide localized alerts in near real time on plume location and movement. A new alert protocol is suggested to help reduce public exposure to pollutants, mitigate associated health risks, and encourage behavioral changes to improve air quality.

## 1. Introduction

Recently, concentrations of fine particulate matter (PM_2.5_) and particulate matter (PM_10_) have emerged as major environmental concerns due to their direct and indirect impacts on human health. Short-term exposure to high levels of PM_2.5_ has been reported to exacerbate respiratory and cardiovascular diseases, leading to acute adverse health effects [1,2,3]. The World Health Organization (WHO) recommends that 24 h average concentrations should not exceed 15 µg/m^3^ for PM_2.5_ or 45 µg/m^3^ for PM_10_ and proposes interim target 1 values—considered more feasible under current conditions—of 75 µg/m^3^ and 150 µg/m^3^, respectively.

In South Korea, an air pollution advisory for PM_2.5_ and PM_10_ is issued when concentrations exceed 75 µg/m^3^ and 150 µg/m^3^, respectively, for at least two consecutive hours. However, this threshold-based approach has limitations, as it may not enable timely responses to rapidly developing high-pollution events. Consequently, there is growing interest in public alert systems capable of providing real-time air quality information and enhancing public awareness during high-concentration episodes [4,5].

Conventional air quality monitoring has largely relied on fixed monitoring networks operated by national or local government agencies. Although the instruments used in these networks provide high measurement accuracy, they are limited by high installation and maintenance costs, as well as low spatial resolution [6]. For example, the city of Seoul, South Korea, operates 50 air quality monitoring stations (AQMSs) across an area of 605 km^2^, consisting of 25 urban stations, 15 roadside stations, and 10 elevated/forest boundary stations. This corresponds to an average spatial coverage of approximately 12 km^2^ (roughly 3.5 km × 3.5 km) per station. Such low spatial resolution makes it difficult to capture localized air pollution events, particularly considering Seoul’s population of approximately 9.5 million.

Meanwhile, many urban studies have employed low-cost sensor networks to expand spatial coverage and obtain meaningful environmental data [7,8]. For instance, López-Restrepo et al. [9] deployed a network of 255 low-cost sensors in the Aburrá Valley, Colombia, to monitor PM_2.5_ concentrations and improve modeling accuracy through data assimilation. Hassani et al. [10] installed 28 low-cost sensors in Kristiansand, Norway, to monitor particulate emissions associated with residential wood burning. In Italy, Penza et al. [11] reported the use of a low-cost sensor network as part of a citizen-participatory air quality monitoring system. In the United States, Barkjohn et al. [12] developed calibration algorithms to improve the accuracy of PurpleAir sensors and demonstrated the reliability of low-cost sensor monitoring by utilizing more than 2000 outdoor PurpleAir sensors across the country.

In South Korea, citizen-driven air quality monitoring services are also in operation, allowing individuals to register privately owned low-cost sensors; the collected data are analyzed in real time and delivered to subscribers [13].

The Smart Seoul Data of Things (S-Dot) utilized in this study is an Internet-of-Things (IoT)-based urban sensor network established in 2019 and operated by the Seoul Metropolitan Government to collect real-time urban environmental data. Previous applications of S-Dot include temperature-based quality control for a meteorological sensor network [14] and integrated analyses linking S-Dot observations with urban environmental, traffic, roadway, and land-use characteristics [15]. Real-time S-Dot measurements are publicly accessible through the Air365 platform (www.air365.co.kr), and detailed documentation is available in Air365 [13]. However, despite its high spatial resolution and real-time environmental sensing capability, no studies have yet examined the use of S-Dot data for detecting localized high-PM_2.5_ or PM_10_ pollution events or for issuing real-time air quality alerts.

In this study, we demonstrate—using the 2023 Seoul International Fireworks Festival case [14,16]—that a low-cost, high-resolution IoT sensor network such as S-Dot can detect short-term localized high-concentration pollution events (local hotspots) in real time, provide information on their spatial distribution and movement to the public, and automatically issue alerts. By doing so, the system can encourage voluntary protective actions by citizens, such as adjusting travel routes, closing windows, or operating air purifiers, thereby reducing exposure to air pollutants. The scientific novelty of this study lies not in the development of the S-Dot network itself but in the useful application of the existing high-resolution S-Dot data for localized PM hotspot detection, AQMS-based field correction of low-cost sensor measurements, plume transport analysis, and preliminary alert protocol development for short-term high-concentration PM events. Furthermore, this study aims to explore strategies for air quality management, exposure reduction, and public health protection in densely populated urban areas.

Numerous studies have reported that fireworks displays exert a substantial impact on particulate matter (PM) concentrations. During the Chinese New Year period, fireworks activities caused sharp increases in PM_10_ and heavy metal concentrations [17]. In Slovenia, PM_2.5_ levels during the 2017 New Year celebration exceeded the January monthly average by more than a factor of ten [18]. In the United States, PM_2.5_ concentrations near fireworks during Independence Day events have been reported to reach approximately 500 µg/m^3^ within an hour [19]. In general, PM_2.5_ and PM_10_ concentrations increase by a factor of 2–14 during fireworks festivals, accompanied by substantial rises in metallic and inorganic particle concentrations [18,20].

In South Korea, average PM_2.5_ concentrations in Seoul and Busan were found to be 7–12 times higher following the 2023 fireworks festival compared with non-event periods [16]. Specific chemical components of PM_2.5_ also increase markedly during fireworks events. Firework-related ions such as K^+^ and Cl^−^ have been reported to account for approximately 28–38% of total PM_2.5_ mass [21]. Furthermore, nitrate (NO_3_^−^) and ammonium (NH_4_^+^) concentrations peak immediately after fireworks events, indicating the influence of precursors generated during the fireworks.

In addition, particulate matter emitted during fireworks displays has been reported to exhibit higher toxicity than ambient background particles. Hickey et al. [22] found that PM_10_ particles originating from fireworks contained elevated concentrations of metals such as lead (Pb), copper (Cu), and barium (Ba). In some samples, lead accounted for approximately 4% of the total PM_10_ mass, while copper accounted for about 1.2%, indicating a notably high metal content. These metal components can promote the generation of reactive oxygen species (ROS), leading to pulmonary inflammation and cellular damage. This evidence suggests that fireworks-derived particulate matter can pose significant biological risks to human health, even with short-term exposure.

The alert alarm protocol utilizing S-Dot, which was applied as an example case in this study to the Seoul International Fireworks Festival case, is expected to be extendable as an automated public air quality notification service for other short-term localized “hotspot” pollution events, such as those caused by fires, industrial explosions, or traffic accidents.

## 2. Materials and Methods

### 2.1. Study Area

**Figure 1:**
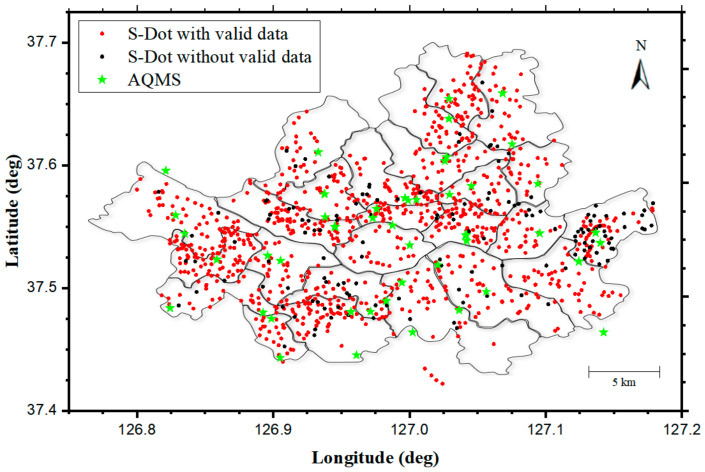
Spatial distribution of S-Dot sites (1138 sites) and AQMSs in Seoul at 21:00 on 7 October 2023. S-Dot sites with valid data signals are shown in red (991 sites, 87% of the total), and stations without data signals are shown in black (147 sites, 13% of the total). A total of 50 AQMSs (green stars) are operated across the city.

As shown in Figure 1, S-Dot is an existing IoT-based urban sensor network operated by the Seoul Metropolitan Government and consists of approximately 1100 outdoor air quality monitors (K-Weather Co., Ltd., Seoul, Republic of Korea) across Seoul. Each monitoring site measures various atmospheric environmental parameters every two minutes, including PM_2.5_ and PM_10_ concentrations, temperature, relative humidity, wind direction, wind speed, ozone (O_3_), and carbon monoxide (CO) [14]. PM_2.5_ and PM_10_ are measured using a low-cost optical particle sensor (PM3006, Cubic Sensor and Instrument Co., Wuhan, China), and the detailed specifications are summarized in Table 1. To mitigate humidity effects, a heater is installed at the PM sensor inlet as described later. The S-Dot sites are serviced on a monthly basis, including sensor replacement and internal cleaning when necessary. For this study, two-minute interval S-Dot data were obtained from the IoT City Data System team of the Seoul Metropolitan Government’s Digital Policy Division. The percentage of S-Dot sites with available 1 h average PM concentrations for the week from 2 to 8 October 2023 was approximately 78%.

The 50 AQMSs operated by the Seoul Metropolitan Government are primarily installed on building rooftops (approximately 15 ± 2 m from the ground) or along major roadways (approximately 4 ± 1 m from the ground). The PM_2.5_ and PM_10_ instruments used in AQMS are beta attenuation monitors, which satisfy performance criteria required by the U.S. Environmental Protection Agency’s Federal Reference Method (FRM), including a multiplicative bias of 0.9–1.1, an additive bias of ±2 µg/m^3^, and a correlation coefficient of ≥0.95. As a result, AQMS measurements are typically within ±10% of the reference values [25]. A specification of the beta attenuation monitor at a representative AQMS is shown in Table 1, which is one of the high-precision instruments registered under the Ministry of Environment, Republic of Korea (MOE)’s certification and approval program [26]. Although these AQMSs provide high measurement accuracy, their low spatial resolution and hourly data availability limit their ability to monitor residents’ real-time exposure levels during short-term, localized high-pollution events. In contrast, S-Dot sensors are installed across representative urban micro-environments—including dense urban areas (87%), areas near mountains or parks (4%), and river-adjacent regions (9%)—allowing for the detection of pollutant exposure under more realistic environmental conditions.

In this study, we evaluated the applicability of the S-Dot network, focusing particularly on short-term, localized high-concentration particle pollution events that occur in large metropolitan cities such as Seoul. We also aimed to explore how useful such high-resolution data is in reducing citizens’ exposure to air pollutants and protecting public health. Among various short-term local pollution events—including fires, explosions, and fireworks events—we selected a representative case for analysis: a fireworks festival in October 2023.

**Figure 2:**
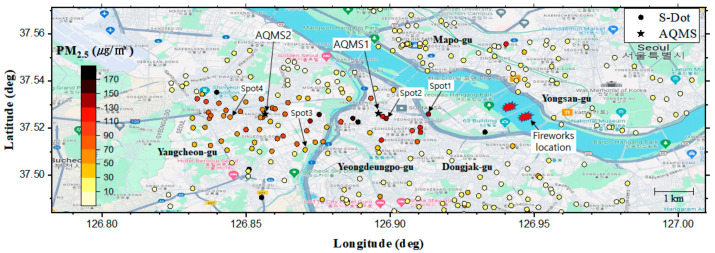
Maximum PM_2.5_ concentrations recorded by S-Dot sites during the fireworks festival (19:00–23:00 on 7 October 2023) without correction, measured at 2 min intervals for the S-Dot and at 5 min intervals for the air quality monitoring stations (AQMSs). AQMS1 is the Yeongdeungpo-gu AQMS, and AQMS2 is the Yangcheon-gu AQMS. Four S-Dot sites (Spot1~Spot4) are chosen to analyze plume transport later.

The 2023 Seoul International Fireworks Festival was held on Saturday, 7 October 2023, from 19:00 to 21:00. Figure 2 shows the locations of the surrounding S-Dot sites and AQMSs, as well as the maximum PM_2.5_ concentrations observed during the event. The highest 2 min average concentrations of PM_2.5_ and PM_10_ were recorded as 180 µg/m^3^ and 250 µg/m^3^ at locations 5.27 km and 1.33 km west of the launch site, respectively. Compared to the October 2023 monthly averages (16 µg/m^3^ for PM_2.5_ and 29 µg/m^3^ for PM_10_), these peak values represent significant increases of 1025% and 762% [27], highlighting the intense short-term impact of the fireworks event. This suggests that the emitted particles at high altitude were transported westward under the influence of easterly wind components.

### 2.2. PM Data Correction for S-Dot Data

Measurement data reliability of low-cost optical particle sensors might depend on particle refractive index, density, morphology, concentration ranges, relative humidity, and so on. Therefore, data correction using reference-grade measurements is needed to ensure reliable results [28].

#### 2.2.1. S-Dot Sensor Specification and Configuration

**Figure 3:**
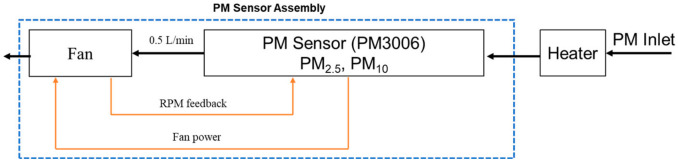
Internal configuration of the S-Dot. Modified from K-Weather [29].

To minimize the influence of relative humidity on particulate matter measurements, S-Dot is equipped with an inlet heater at the sampling inlet (Figure 3).

For the correction of S-Dot measurements, PM_2.5_ and PM_10_ data from the nearest urban AQMS were compared. For example, one S-Dot site (Site ID: V02Q1940850) is located at 37.5250° N, 126.8974° E, while its nearest urban AQMS, the Yeongdeungpo AQMS (AQMS1 in Figure 2), is located at 37.5263° N, 126.8960° E, resulting in a separation distance of approximately 191 m. PM_2.5_ and PM_10_ concentrations at the 50 AQMS sites operated by the Seoul Metropolitan Government are measured using beta attenuation monitors made by a few different manufacturers. The specifications of the PM sensor of the S-Dot network and the beta attenuation monitor of AQMS1 are summarized in Table 1.

#### 2.2.2. Delay Time Correction for AQMS Timestamps

**Figure 4:**
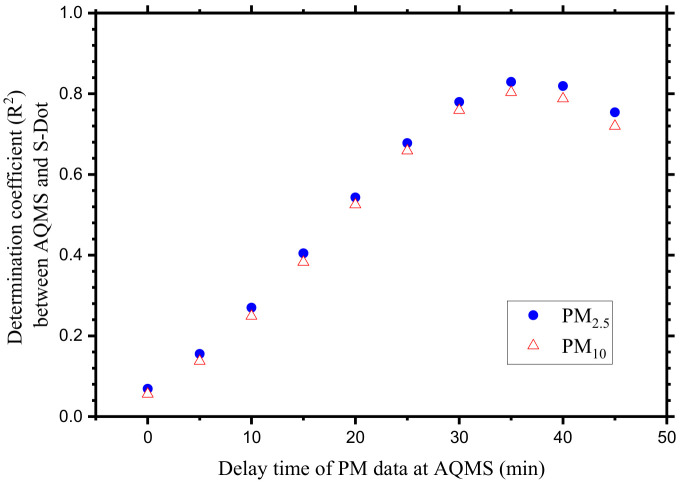
Determination coefficients (R^2^) between AQMS1 and the nearest S-Dot site for PM_2.5_ and PM_10_ (19:00~23:00 on 7 October 2023) under varying AQMS1 delay times in 5 min intervals (0–45 min). The determination coefficient exhibits a distinct maximum when the delay time of the PM data at the AQMS is assumed to be 35 min, which suggests that the temporal alignment between the AQMS and S-Dot data is optimized at this delay for AQMS data.

Because the temporal resolutions of the AQMS data and S-Dot data used in this study are 5 min and 2 min, respectively, both datasets were averaged to 10 min intervals. As shown in Figure 4, the coefficient of determination (R^2^) between the nearest S-Dot measurement and the corresponding AQMS1 measurement was below 0.2, indicating very poor agreement. Due to the characteristics of the beta-gauge measurement and data-processing algorithm, PM data at the AQMS can exhibit response delays of a few tens of minutes. To account for this potential delay, AQMS timestamps were shifted by assuming delay times ranging from 5 to 40 min, in 5 min increments, and R^2^ was recalculated for each scenario. As illustrated in Figure 4, the highest R^2^ value was obtained when a 35 min delay was assumed. Therefore, the AQMS data were considered to have a 35 min response delay, and their timestamps were corrected by subtracting 35 min from the original time.

**Figure 5:**
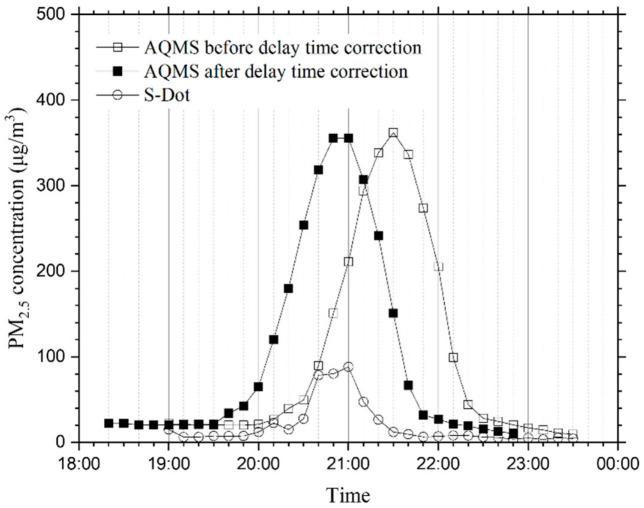
PM_2.5_ concentrations measured at AQMS1 and the nearest S-Dot site on 7 October 2023, before and after applying the 35 min delay time to the AQMS1 timestamps. A similar result for PM_10_ concentrations is shown in Figure S3.

Figure 5 compares the temporal variations in S-Dot PM_2.5_ concentrations with AQMS measurements before and after applying the time-shift correction. After correcting AQMS timestamps using 35 min delay time, the peak timings of AQMS1 and the nearest S-Dot measurements were found to align closely. This delay time of the AQMS1 was also applied to the other urban AQMSs’ data. However, it should be noted that the exact delay time at the other urban AQMSs might not be the same as that of the AQMS1 because of differences in manufacturers and/or instrument conditions, and collocated intercomparison studies are needed in the future.

#### 2.2.3. Real-Time PM Concentration Correction for S-Dot Data

For each S-Dot site, the PM_2.5_ and PM_10_ concentrations were corrected using the data from the nearest AQMS. For PM_2.5_, a linear regression equation (y = ax + b) was derived using 10 min averaged concentrations from 35 min to 4 h prior to the correction time, and this regression was then applied to adjust the S-Dot concentrations during the most recent 35 min period. In addition, the PM_2.5–10_ concentration (calculated as PM_10_ minus PM_2.5_) was corrected separately by deriving a regression relationship with the AQMS PM_2.5–10_ values. The corrected PM_2.5–10_ concentration was then added to the corrected PM_2.5_ concentration to obtain the corrected PM_10_ concentration. When the S-Dot PM_2.5–10_ concentration resulted in a negative value, the PM_2.5–10_ concentration was set to zero to prevent inconsistencies in which the corrected PM_10_ concentration becomes lower than the corrected PM_2.5_ concentration. For example, the correction trend line at 15:00 was derived using the data from 10:30 to 14:30, and this trend was used to correct the concentrations measured between 14:30 and 15:00. During the period of elevated PM_2.5_ and PM_10_ concentrations associated with the fireworks event on 7 October 2023, the R^2^ values between AQMS and S-Dot measurements exceeded 0.8, indicating strong correlations. Although the R^2^ values were relatively low during periods of low pollution, the differences between corrected and uncorrected PM_2.5_ and PM_10_ concentrations were negligible (Figure S5). Therefore, the lower correlation in clean periods had minimal impact on identifying local high-concentration pollution events (hotspots).

**Figure 6:**
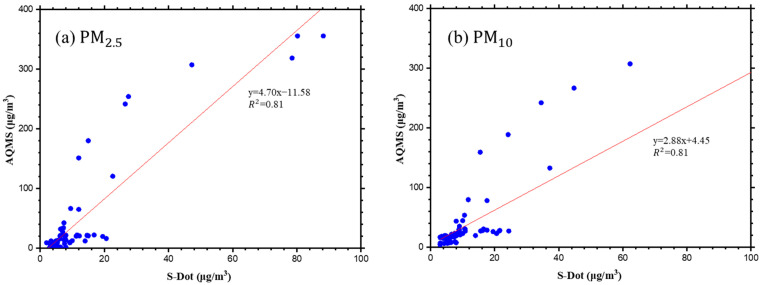
Correlation between AQMS1 and the nearest S-Dot site (V02Q1940850) based on 10 min averaged PM_2.5_ (**a**) and PM_10_ (**b**) data during the fireworks festival on 7 October 2023.

As shown in Figure 6, the comparison between the PM_2.5_ and PM_10_ concentrations measured by the S-Dot sensor nearest to AQMS1 during the fireworks event yielded R^2^ values of 0.81, indicating relatively strong correlations. For real-time correction of the S-Dot data using AQMS measurements as a reference, a linear trend line was derived from the PM concentration data collected over the preceding four hours prior to the latest measurement. This regression was then applied to adjust the most recent S-Dot sensor concentrations. Although a quadratic regression resulted in a slightly greater coefficient of determination (R^2^ = 0.83), linear regression was chosen in this study for a more robust correction because regression functions can vary considerably depending on the characteristics of individual high-concentration pollution episodes, potentially reducing the consistency of the correction approach across different events.

## 3. Results

**Figure 7:**
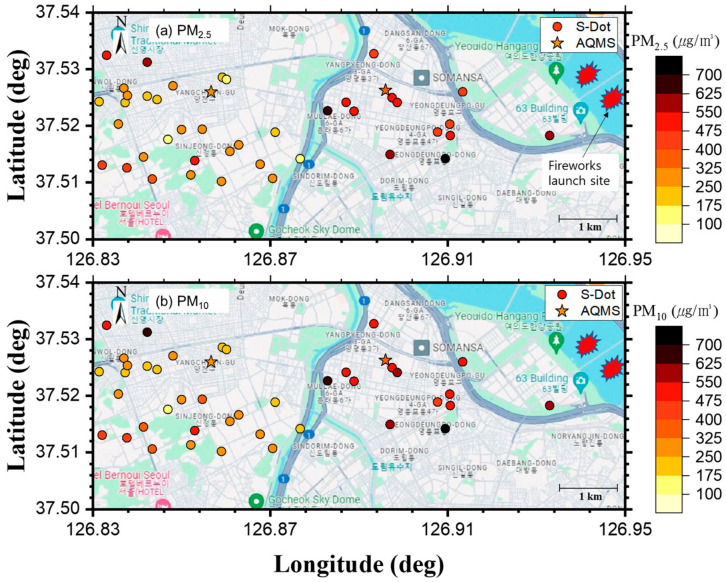
Maximum PM_2.5_ (**a**) and PM_10_ (**b**) concentrations measured by S-Dot sites during the fireworks festival (19:00–23:00) after the data correction. The red burst symbols indicate the fireworks launch sites. Four high-concentration sites (Spot1–Spot4) mark the path of a fine dust plume that was emitted from the festival site and moved westward.

Figure 7 presents the spatial distribution of the peak PM_2.5_ and PM_10_ concentrations during the fireworks event after applying the S-Dot data correction. Elevated levels of both PM_2.5_ and PM_10_ were observed extending westward from the festival site.

**Figure 8:**
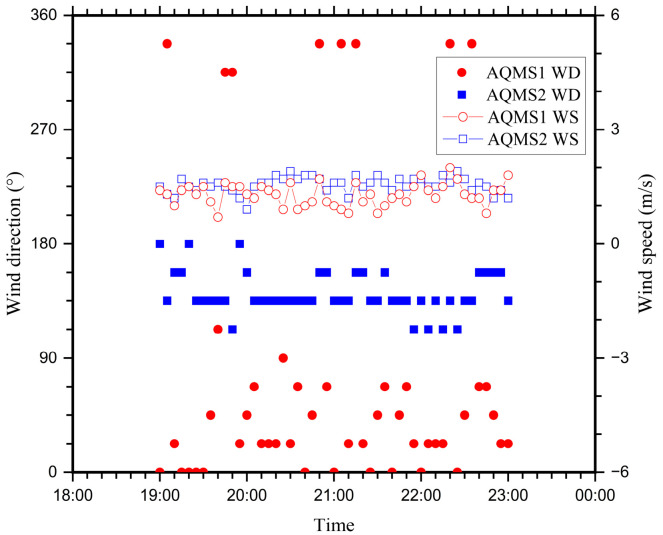
Wind direction and wind speed measured at AQMS1 and AQMS2 during the fireworks festival (19:00–23:00).

As shown in Figure 8, which presents the wind direction and wind speed data from AQMS1 (located approximately 12.3 m above ground level) and AQMS2 (located approximately 16.5 m above ground level), easterly winds predominated during the fireworks event. The wind speed ranged from 0.7 to 1.9 m/s, which was characterized by relatively weak winds or nearly calm conditions with limited dilution capacity, thereby providing favorable conditions for pollutant transport during the entire fireworks period (Figure 8). At 21:00 on 7 October 2023, the Yeongdeungpo area recorded a temperature of approximately 19.0 °C, relative humidity of 57%, wind direction from the north-northeast, and wind speed of 0.7 m/s [30]. Regional-scale near-surface wind fields over the Korean Peninsula (Figure S6) also showed prevailing easterly flow. The backward trajectory of the air mass arriving at Spot4 in the late evening after fireworks events shows the movement of air from east to west within the Seoul area (Figure S7). Under these meteorological conditions, PM_2.5_ emitted from the festival site was likely transported westward, dispersing through Yeongdeungpo-gu toward Yangcheon-gu. The satellite-derived aerosol optical depth (AOD) data from the MODIS/Terra did not indicate significant aerosol transport into the Seoul area during the fireworks period (Figure S8). Therefore, the elevated PM concentrations observed during the fireworks festival were considered to be primarily associated with local emissions from the fireworks rather than long-range transboundary pollution transport across borders.

**Figure 9:**
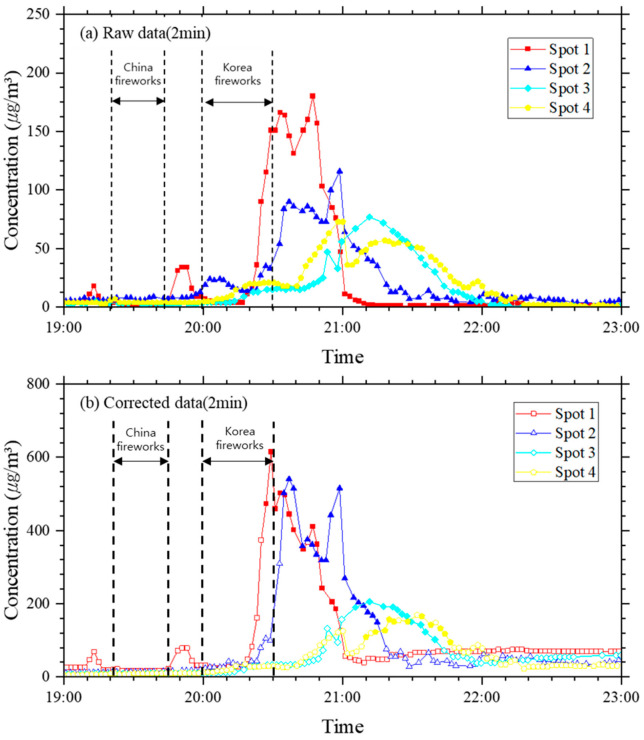
PM_2.5_ concentrations measured at S-Dot sites during the fireworks festival (19:00–23:00 on 7 October 2023) (**a**) before and (**b**) after correction. The vertical dashed lines indicate the duration of the fireworks performance by Team China and Team Korea. In panel (**b**), filled symbols represent instances when alerts were issued.

The fireworks festival began at 19:20 with a performance by the Chinese team, followed by the Polish team, which was scheduled at 19:40. However, due to unforeseen circumstances, the Polish team’s display did not take place. The Korean team subsequently conducted the final show from 20:00, which concluded at approximately 20:30. Figure 9a presents the variation in PM_2.5_ concentrations observed at Spot 1, the monitoring site closest to the festival location (1.33 km distance). A small peak was detected before 20:00, and a high-concentration period lasting approximately 30 min was observed beginning at around 20:30. About 20 min after each national team started their fireworks, the concentration at Spot 1 began to increase rapidly. This delay is accounted for by the transport time due to wind. After applying the correction (Figure 9b), the highest PM_2.5_ concentrations appeared sequentially from the monitoring sites closest to the festival venue, with concentrations gradually decreasing with increasing distance. These findings indicate that PM_2.5_ levels diminished as the plume dispersed westward from the festival site.

## 4. Discussion

S-Dot has the potential to evolve from a simple alerting tool into a citizen-driven environmental monitoring platform, providing valuable data to support city-scale air quality management strategies. Currently, the city of Seoul issues air pollution advisories and warnings when the hourly average PM_10_ concentrations exceed 150 µg/m^3^ and 300 µg/m^3^, respectively, at all 25 AQMSs for two consecutive hours. For PM_2.5_, the thresholds are 75 µg/m^3^ and 150 µg/m^3^ [26]. In 2023, Seoul issued eight PM advisories and no warnings.

**Figure 10:**
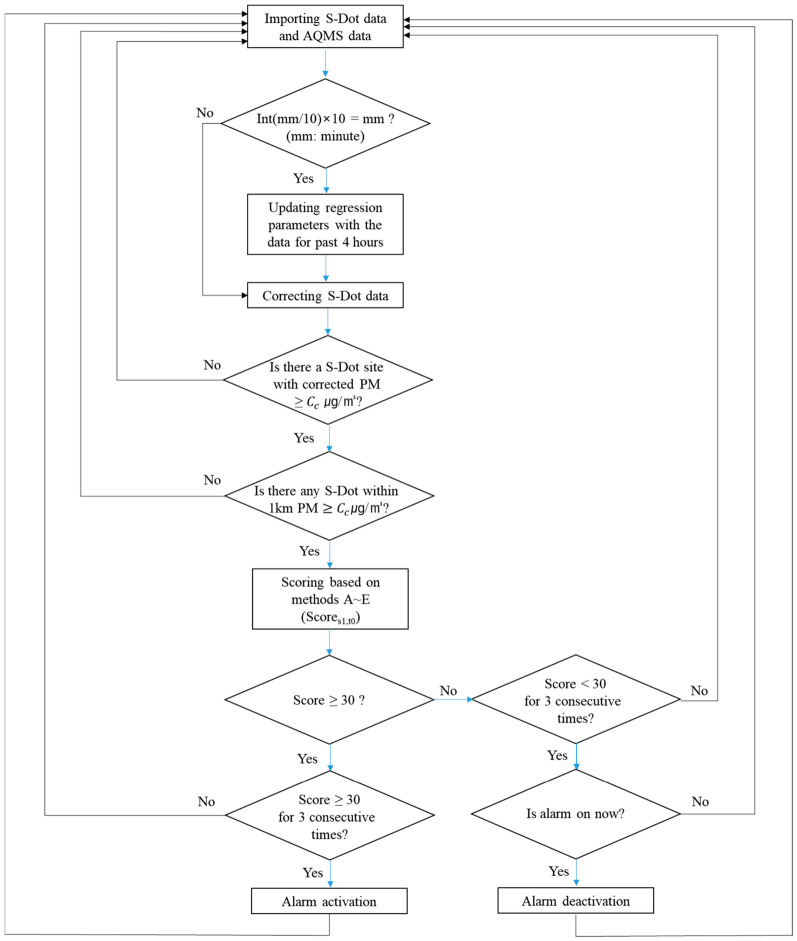
Flowchart of the proposed alarm protocol integrating S-Dot and AQMS data. The process includes real-time data correction based on 4 h regression parameters, spatial cross-verification within a 1 km radius, and a scoring-based decision matrix for alarm activation and deactivation. (Threshold concentration, C_c_: 75 µg/m^3^ for PM_2.5_, 150 µg/m^3^ for PM_10_).

During the 2023 Seoul International Fireworks Festival on 7 October 2023, PM_2.5_ concentrations exceeded 75 µg/m^3^ for approximately one hour across many locations. However, these short-term spikes did not meet the official criteria for public alerts, and thus no government-issued warning was released. Figure 10 illustrates the proposed alert protocol designed to issue warnings when PM_2.5_ exceeds 75 µg/m^3^ or PM_10_ exceeds 150 µg/m^3^, conditions under which potential health risks may arise. First, S-Dot measurements are corrected using the derived field correction equations. Next, each S-Dot site is evaluated to determine whether PM_2.5_ concentrations exceed 75 µg/m^3^ or PM_10_ concentrations exceed 150 µg/m^3^. Then, it is verified whether nearby S-Dot monitors located within a 1 km radius also exceed the same thresholds. For the S-Dot sites that meet these criteria, a score is calculated by summing five evaluative components:

(1) Score=A+B+C+D+E

where

(2) $$A=\frac{{PM}_{2.5,10}\;}{10}$$

(3) $$B=\frac{{PM}_{2.5}\left(t\right)-{PM}_{2.5}\left(t-1\right)}{{PM}_{2.5}\left(t-1\right)}\times \left(\frac{{PM}_{2.5}}{{PM}_{10}}\times 2\right)\times 20$$

(4) $$C=(n-1)\times int(\frac{{PM}_{2.5,10}}{100})$$

(5) $$D=N_{s}\times 5$$

(6) $$E=10\times int(\frac{x}{100})$$

Here, PM_2.5,10_ represents the PM_2.5_ concentration (µg/m^3^) measured at the corresponding S-Dot monitoring site, and PM_10_ represents the PM_10_ concentration (µg/m^3^) at the same site. *n* denotes the number of consecutive occurrences in which PM_2.5_ exceeded 75 µg/m^3^ or PM_10_ exceeded 150 µg/m^3^. *N_s_* refers to the number of S-Dot monitors within a 1 km radius where PM_2.5_ concentrations exceeded 75 µg/m^3^ or PM_10_ exceeded 150 µg/m^3^. *x* represents the PM_2.5_ or PM_10_ concentration measured by an AQMS located within a 1 km radius that also exceeded the respective thresholds.

In determining the score, weighting factors are applied to incorporate the concentrations measured at nearby S-Dot sites and AQMSs. This approach helps prevent false alarms triggered by highly localized, short-lived pollution events—such as those caused by cigarette smoke—by ensuring that an alert is issued only when elevated pollution levels are observed consistently across neighboring sensors.

**Figure 11:**
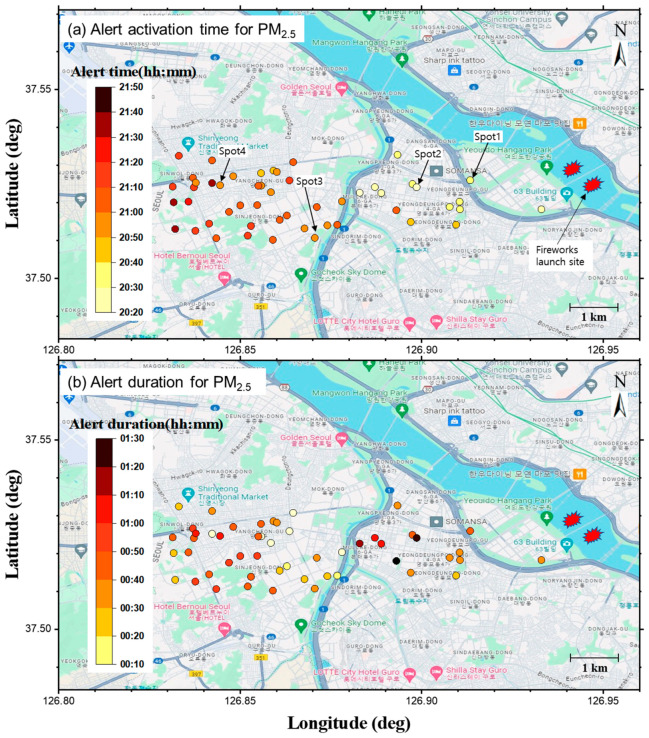
Application examples of the new alarm protocol using the fireworks case on 7 October 2023. (**a**) Alert activation time for PM_2.5_; (**b**) alert duration for PM_2.5_. The red burst symbols indicate the fireworks launch sites. The same results for PM_10_ are shown in Figure S10 of the Supporting Information.

An alert is issued when the score exceeds 40 for three consecutive evaluations, and the alert is lifted when the score falls below 40 for three consecutive evaluations. If the proposed alert protocol had been applied during the fireworks display, alerts would have been triggered in areas that closely matched the predicted spatial pathway of PM_2.5_ dispersion (Figure 11). By providing high-resolution, bias-corrected data at 2-min intervals, S-Dot can effectively complement the limited temporal and spatial resolution of the existing air quality monitoring network in Seoul. This enables real-time alerts for short-lived, localized high-concentration PM events and allows the rapid communication of micro-scale pollution episodes that would otherwise remain undetected by the current alert system, thereby helping to reduce public exposure.

## 5. Conclusions

In this study, we analyzed localized high-concentration PM_2.5_ and PM_10_ pollution that occurred during the Seoul Fireworks Festival using the S-Dot sensor network data and proposed an alert protocol capable of real-time response. The existing air quality monitoring network issues advisories and warnings based on 2 h average concentrations across Seoul, which limits its ability to capture short-term and spatially localized high-pollution events. In contrast, S-Dot provides minute-level measurements, enabling much finer temporal and spatial resolution. For concentration correction, each S-Dot sensor was individually paired with the nearest AQMS, and correction coefficients were derived and applied to the corresponding S-Dot data. Given the characteristics of low-cost sensors and the spatial variability inherent to their measurements, this approach represents the most feasible correction method currently available. The goal of the correction process was not to achieve perfect accuracy in absolute concentration levels but rather to identify the location and duration of local hotspot events so that the information can be used effectively for public alerts.

The alert scoring system was designed to incorporate both the corrected S-Dot data and AQMS measurements, while accounting for PM_2.5_/PM_10_ fractions and the duration of elevated concentrations. In this study, the alert score was computed based on factors such as the rate of concentration increase, persistence of high concentrations, and PM composition characteristics. Weighting factors were assigned by considering the sensitivity and relative influence of each component (e.g., higher weights were applied to short-term concentration gradients and the proportion of PM_2.5_). This scoring framework not only allows for the rapid detection of localized plumes but also has the potential to trigger meaningful behavioral responses among citizens. Timely alerts can help individuals who may be exposed to the plume adjust their movement paths, close windows, or operate air purifiers, thereby reducing potential exposure.

Applying the proposed protocol to the fireworks festival event on 7 October 2023 demonstrated that the system could rapidly identify areas experiencing short-term, high-concentration PM_2.5_ and PM_10_ pollution immediately after the event.

Therefore, despite the inherent limitations of low-cost sensors, the S-Dot-based alert protocol proposed in this study empirically demonstrates the preliminary feasibility of establishing a temporally and spatially refined PM alert system. This approach is expected to complement future real-time urban air quality management frameworks and enhance their responsiveness to localized high-concentration pollution events.

S-Dot has the potential to evolve into a citizen-participatory environmental monitoring platform by leveraging integration with geographic information systems (GISs) and mobile technologies, thereby enhancing accessibility and scalability. Currently, S-Dot employs low-cost sensors that classify particulate matter solely on a mass concentration basis. However, certain PM emission sources—such as fireworks and non-exhaust traffic emissions—often contain substantial fractions of metallic particles. Li et al. [31] developed a low-cost technique capable of measuring metal content, known as spark-induced breakdown spectroscopy (SIBS), which holds potential for future integration into sensor networks. Such technological advancements could extend the applicability of S-Dot beyond concentration monitoring toward real-time source identification and health risk assessment.

In this study, the correction coefficients for S-Dot data were derived from comparisons between each S-Dot site and its nearest AQMS data. However, this correction method is limited by local variations arising from site-specific environmental characteristics and the optical properties of particulate matter [32], and further analyses considering meteorological conditions and multiple emission sources are also necessary [33]. Even though differences in distance, measurement height, and local environmental conditions between paired S-Dot and urban AQMS may contribute to discrepancies that reflect not only sensor bias but also real spatial variability, this field correction method is found useful for the fireworks case. To improve the correction method and the reliability of sensor deployment, it is recommended to strategically install multiple low-cost sensors near AQMS sites, taking into account changes in spatial observation capability due to sensor density and maintenance costs [14,34]. In addition, qualitative studies are required to assess whether high-concentration particulate matter alerts actually influence citizens’ behavioral responses [35,36].

In particular, data gaps arising from maintenance issues of low-cost sensors may lead to underestimation of the spatial extent of high-concentration PM_2.5_. The single linear correction approach applied in this study also has limitations, as it does not fully account for variations in meteorological conditions, optical response characteristics, or humidity. Furthermore, the optimal regression function based on the nearest AQMS data should be investigated in future studies. Although alternative regression functions, such as quadratic functions, may provide improved performance under specific conditions, the most appropriate regression curve is likely to vary depending on pollution characteristics, aerosol composition, and meteorological conditions. Therefore, additional studies encompassing diverse pollution episodes are required to identify the most robust and transferable correction approach. It should also be noted that this is a preliminary study primarily focused on monitoring local hotspots, in other words, high-concentration data, for protecting public health. Previous studies have repeatedly reported that simple linear correction tends to overestimate concentrations and reduce accuracy under high-humidity conditions and have suggested that multiple linear regression, incorporating variables such as temperature and humidity, or machine learning-based correction capable of capturing nonlinearity (e.g., Random Forest, Gradient Boosting) can achieve higher performance. Therefore, to enhance the reliability of S-Dot sensors, revalidation under diverse seasonal, regional, and pollution conditions, together with the application of advanced field correction techniques, is essential.

In addition, while a 4 h window was utilized for regression updates in this study, further investigations are needed to evaluate the effects of different temporal scales (e.g., 2, 6, or 8 h) on the correction results to identify the most effective update interval.

Regarding the alert protocol, the present study should be regarded as a preliminary proof of concept rather than a statistically robust validation of a generalized alert system. Although the proposed protocol was applied to the fireworks event to examine its feasibility, the alert score parameters, such as weighting factors, alert thresholds, and the number of consecutive evaluations, were primarily developed based on the fireworks case without optimization. Therefore, the transferability of the protocol to other types of localized high-concentration PM events remains uncertain. Future studies with a sensitivity analysis should apply this alert protocol to a larger number of independent cases, including fires, industrial explosions, traffic accidents, and regional pollution episodes, and evaluate its performance using quantitative metrics such as detection rate, false alarm rate, missed alarm rate, lead time, and alert duration accuracy. Such validation will be necessary before the protocol can be implemented as a generalized operational alert system.

In addition, predictive models for early detection of high-concentration events, adaptive alert systems based on citizen behavioral responses, and collaborative policy frameworks with local communities and authorities should be developed concurrently. Such follow-up studies are anticipated to substantially enhance the reliability of urban-scale air quality management and the efficiency of protecting public health.

## Figures and Tables

**Table 1:** Specifications of the instruments at AQMS1 and low-cost sensors at the S-Dot sensor network for PM_2.5_ and PM_10_ [23,24].

Site	Yeongdeungpo-gu Air Quality Monitoring Station (AQMS1)	S-Dot Sensor Network (K-Weather OAQ) *
Manufacturer	Kimoto Electric Co., Ltd. (Osaka, Japan)	Cubic Sensor and Instrument Co., Ltd. (Wuhan, China)
Model	PM-711	PM3006
Measuring method	Beta ray attenuation method	Laser scattering
Measuring range	0–5000 μg/m^3^	0–30,000 μg/m^3^
Reliability	Zero drift: ±5 μg/m^3^/day Span drift: ±3%/day of equivalent film number Linearity: ±5% of equivalent film number Reading for standard air sample: ±10% of mass concentration	Accuracy of PM_1_ and PM_2.5_: <35 μg/m^3^: ±5 μg/m^3^; 35~100 μg/m^3^: ±10 μg/m^3^; 100~1000 μg/m^3^: ±10% Accuracy of PM_10_: ≤100 μg/m^3^: ±15 μg/m^3^, 100~1000 μg/m^3^: ±15% at the conditions of 25 ± 2 °C & 50 ± 10% RH (Reference instrument: GRIMM)
Sampling flow rate	16.7 LPM (20 °C, 1 atm)	0.5 LPM
Etc	Sampling filter: PTFE Beta source: 14C (<10 MBq) Detector: Plastic scintillation	Inlet heater temperature: 50 °C Response time: 1 s

* Monitoring parameters of S-Dot: PM_2.5_, PM_10_, temperature, relative humidity, wind speed, wind direction, light intensity, UV, noise level, vibration level, globe temperature, CO, NO_2_, SO_2_, NH_3_, H_2_S, O_3_.

## Data Availability

The original contributions presented in this study are included in the article/Supplementary Materials. Further inquiries can be directed to the corresponding author.

## Supplementary Materials

The following supporting information can be downloaded at https://www.mdpi.com/article/10.3390/s26134307/s1, Figure S1: Maximum PM_10_ concentrations recorded by S-Dot sites during the fireworks festival (19:00–23:00 on 7 October 2023) without correction, measured at 2 min intervals for the S-Dot and at 5 min intervals for the air quality monitoring stations (AQMSs). AQMS1 is the Yeongdeungpo-gu urban AQMS, and AQMS2 is the Yangcheon-gu urban AQMS; Figure S2: Example of an actual S-Dot site installation; Figure S3: PM_10_ concentrations measured at AQMS1 and the nearest S-Dot site on 7 October 2023, before and after applying the 35 min delay time to the AQMS1 timestamps; Figure S4: Representative results of the linear regression for PM_2.5_ and PM_2.5–10_ between AQMS1 and its nearest S-Dot. (a) Slope of the correction equation (y = ax + b) calculated every 4 h from 15:00 on 7 October 2023 to 03:00 on 8 October 2023; (b) y-axis intercept; (c) coefficient of determination (R^2^) between AQMS1 and the nearest S-Dot within each 4 h window; Figure S5: Ten-minute averaged PM concentrations at AQMS1 and its nearest S-Dot site (ID: V02Q1940850) from 15:00 on 7 October 2023 to 03:00 on 8 October 2023. (a) PM_2.5_; (b) PM_2.5–10_; (c) PM_10_; Figure S6: Surface wind visualization around the Korean Peninsula at 23:00 KST on 7 October 2023 [37]. Modified by the authors; Figure S7: Hybrid Single-Particle Lagrangian Integrated Trajectory (HYSPLIT version 5.3.0) analysis results. The 72 h backward trajectories arriving at 100, 250, and 500 m above ground level (AGL) during the fireworks event on 7 October 2023. Trajectories were calculated for the nearest hourly time point to the observed concentration peak at Spot 4 (37.52° N, 126.84° E; 22:00 KST); Figure S8: MODIS/Terra aerosol optical depth (AOD) over East Asia during the study period: (a) 5 October 2023; (b) 6 October 2023; (c) 7 October 2023 (the date of the Seoul International Fireworks Festival); and (d) 8 October 2023. The website is Level-1 and Atmosphere Archive & Distribution system (https://ladsweb.modaps.eosdis.nasa.gov/view-data/ accessed on 1 June 2026). No significant transboundary aerosol transport affecting the Seoul metropolitan area was observed during the fireworks festival period, suggesting that the elevated PM concentrations measured on 7 October 2023 (KST) were unlikely to be influenced by long-range transport from outside Korea; Figure S9: PM_10_ concentrations measured at S-Dot sites during the fireworks festival (19:00–23:00 on 7 October 2023) (a) before and (b) after correction. The vertical dashed lines indicate the duration of the fireworks displays by Team China and Team Korea. In panel (b), filled symbols represent instances when alerts were issued; Figure S10: Application results of the new alarm protocol to the fireworks case on 7 October 2023. (a) Alert activation time for PM_10_; (b) alert duration for PM_10_. The red burst symbols indicate the fireworks launch sites.
