# Supplementary material for: Application of Stationary IoT Sensor Networks to Reduce Exposure to Particulate Matter During Local Hotspot Events in a Megacity: A Fireworks Case Study
Source: Sensors (Basel). 2026 Jul 7;26(13):4307. doi: 10.3390/s26134307 (PMC13363929; doi:10.3390/s26134307)
Supplement: Supplementary file 1 [file sensors-26-04307-s001.zip › sensors-4349508-supplementary.pdf]

Supporting Information

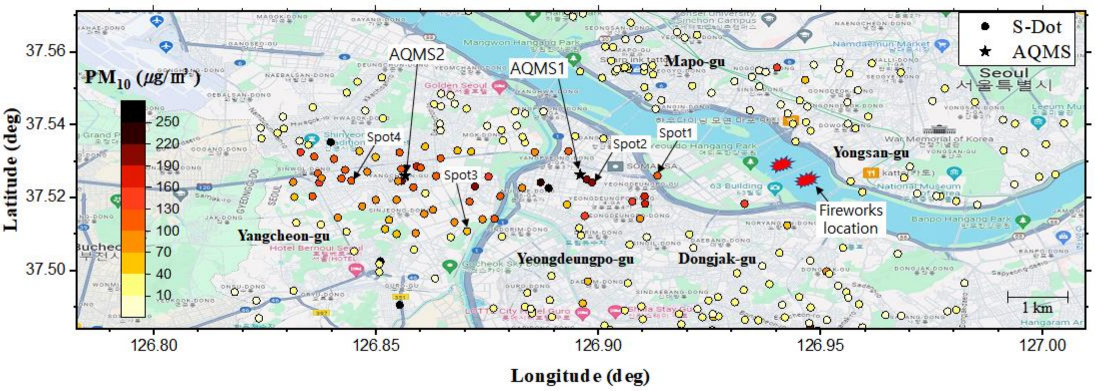

Figure S1. Maximum PM10 concentrations recorded by S-Dot sites during the fireworks festival (19:00–23:00 on 7 October 2023) without correction, measured at 2-minute intervals for the S-Dot and at 5-minute intervals for the air quality monitoring stations (AQMSs). AQMS1 is the Yeongdeungpo-gu urban AQMS, and AQMS2 is the Yangcheon-gu urban AQMS.

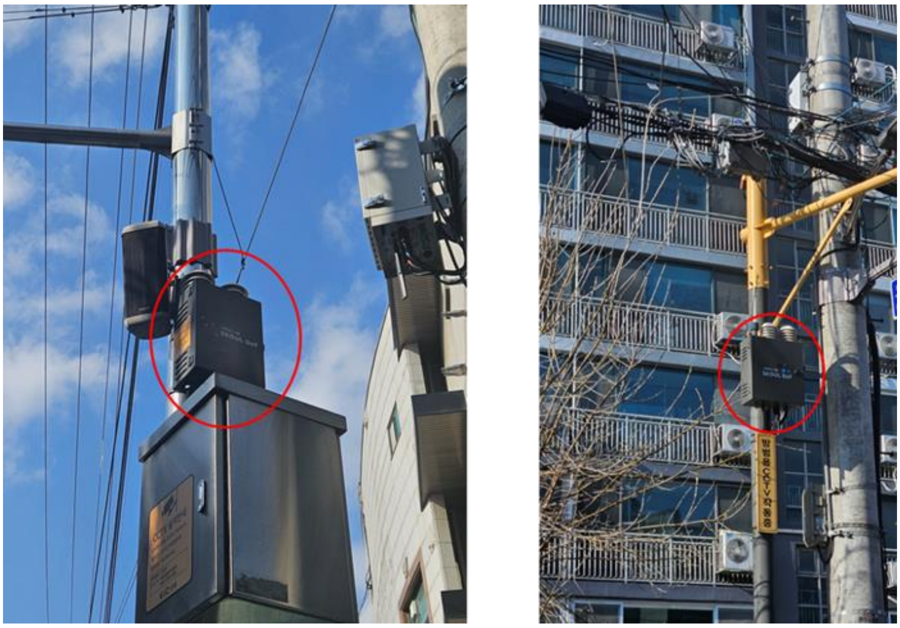

Figure S2. Example of an actual S-Dot site installation.

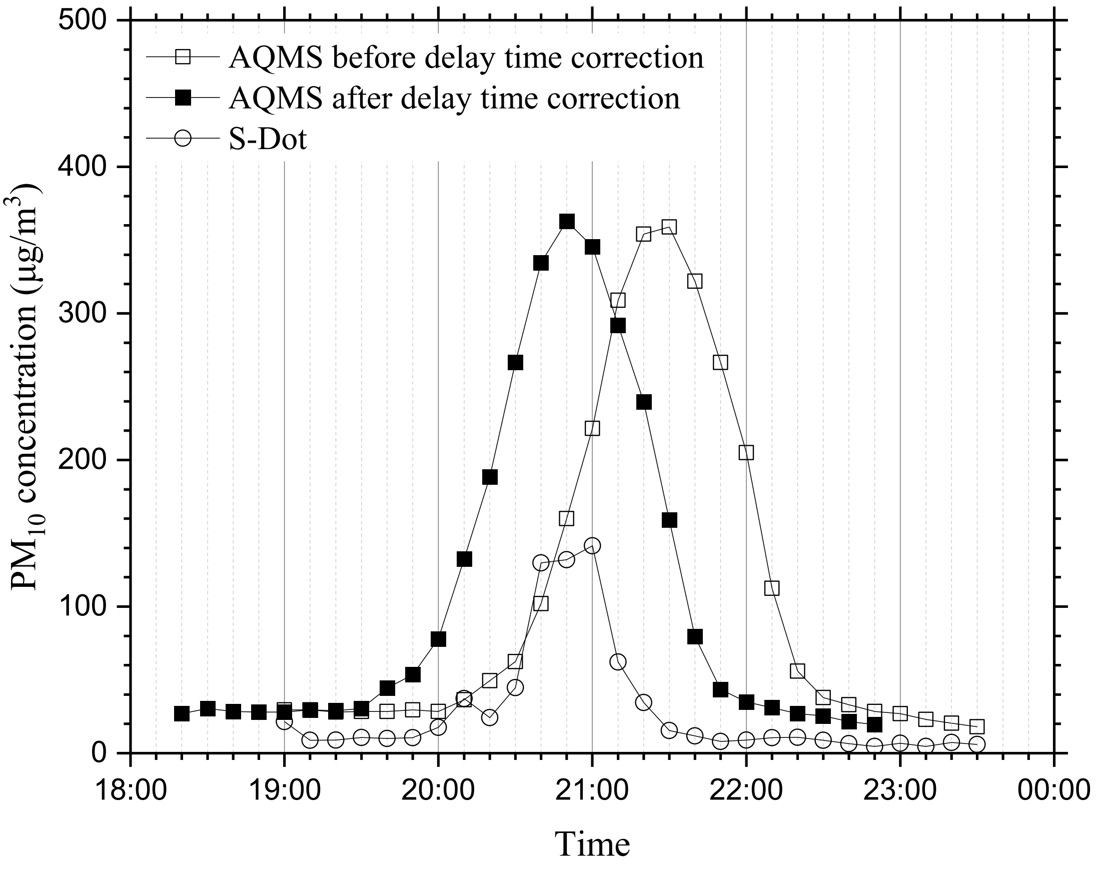

Figure S3. PM_{10} concentrations measured at AQMS1 and the nearest S-Dot site on 7 October 2023, before and after applying the 35-minute delay time to the AQMS1 timestamps.

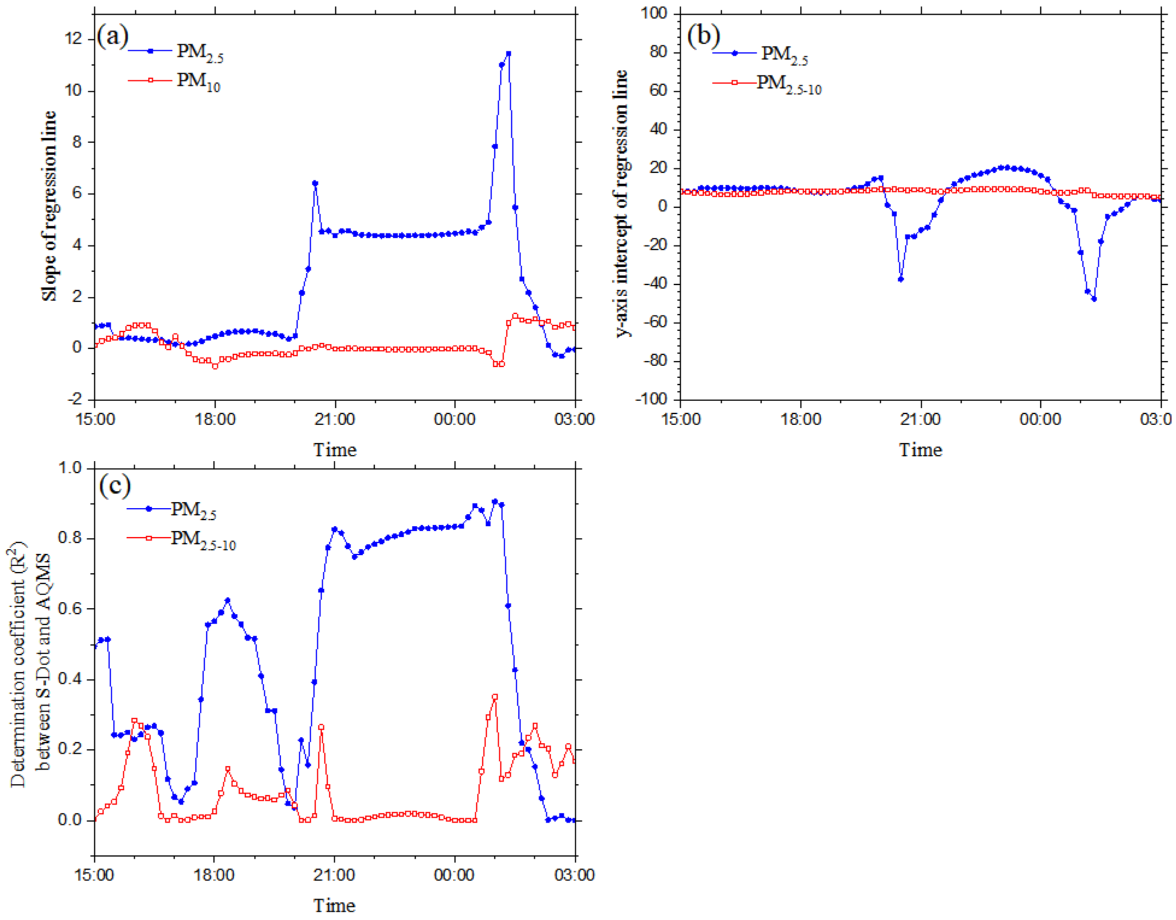

Figure S4. Representative results of the linear regression for $PM_{2.5}$ and $PM_{2.5\text{-}10}$ between AQMS1 and its nearest S-Dot. (a) Slope of the correction equation ($y = ax + b$) calculated every 4 h from 15:00 on 7 October 2023, to 03:00 on 8 October 2023; (b) y-axis intercept; (c) coefficient of determination (R^2) between AQMS1 and the nearest S-Dot within each 4 h window.

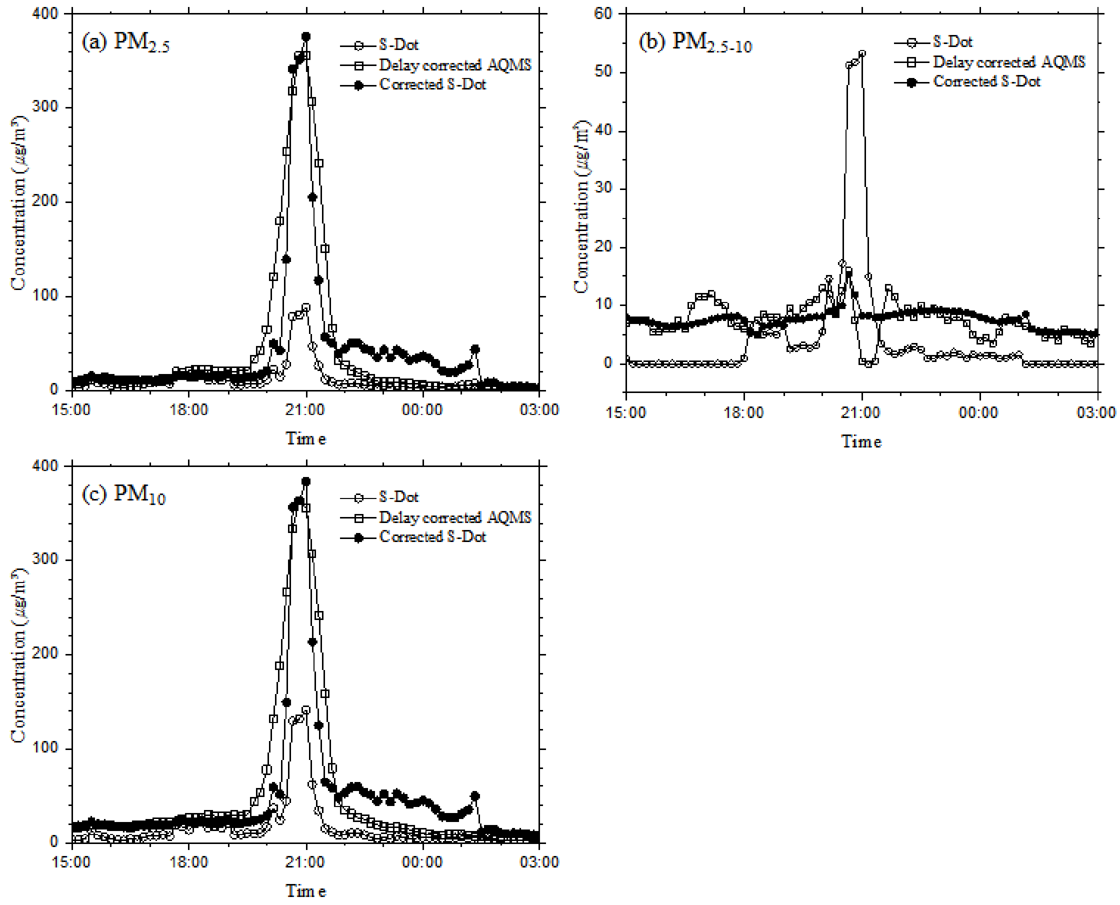

Figure S5. Ten-minute averaged PM concentrations at AQMS1 and its nearest S-Dot site (ID: V02Q1940850) from 15:00 on 7 October 2023, to 03:00 on 8 October 2023. (a) $PM_{2.5}$, (b) $PM_{2.5\text{-}10}$, (c) PM_{10}.

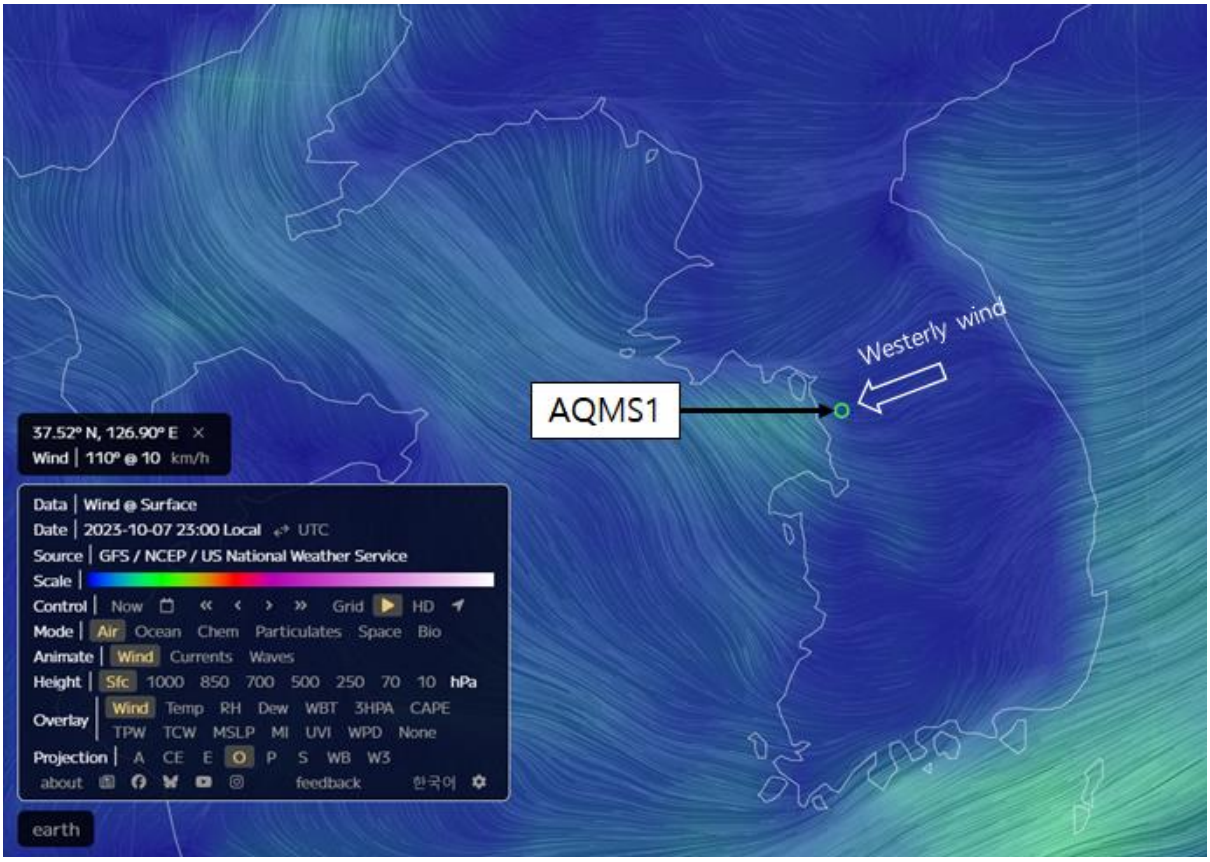

Figure S6. Surface wind visualization around the Korean Peninsula at 23:00 KST on 7 October 2023 [37]. Modified by the authors.

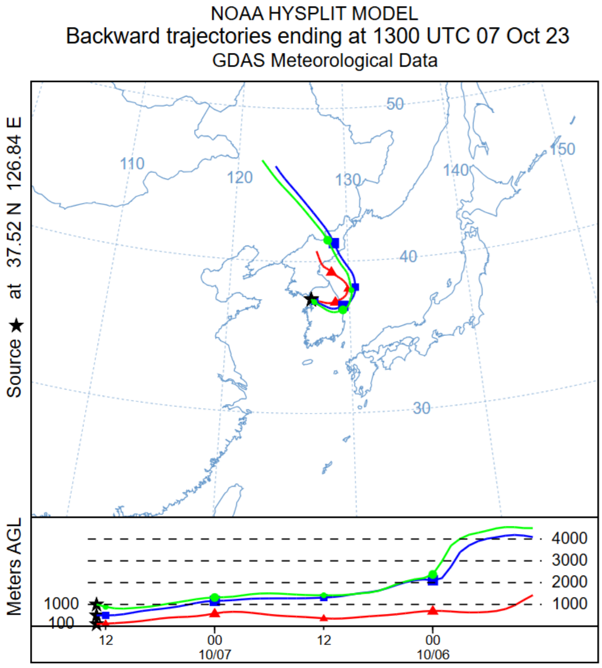

Figure S7. Hybrid Single-Particle Lagrangian Integrated Trajectory (HYSPLIT version 5.3.0) analysis results. The 72 h backward trajectories arriving at 100, 250, and 500 m above ground level (AGL) during the fireworks event on 7 October 2023. Trajectories were calculated for the nearest hourly time point to the observed concentration peak at Spot 4 (37.52° N, 126.84° E; 22:00 KST).

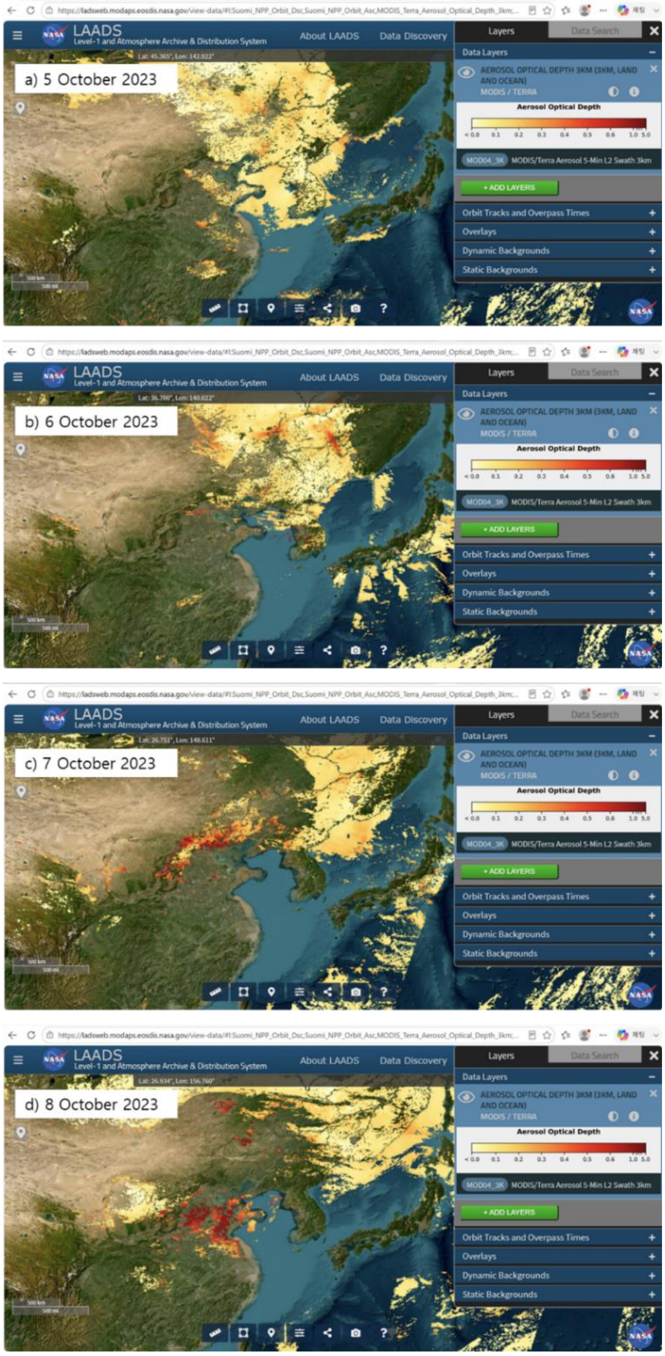

Figure S8. MODIS/Terra aerosol optical depth (AOD) over East Asia during the study period: (a) 5 October 2023; (b) 6 October 2023; (c) 7 October 2023 (the date of the Seoul International Fireworks Festival); and (d) 8 October 2023. The website is Level-1 and Atmosphere Archive & Distribution system (https://ladsweb.modaps.eosdis.nasa.gov/view-data/). No significant transboundary aerosol transport affecting the Seoul metropolitan area was observed during the fireworks festival period, suggesting that the elevated PM concentrations measured on 7 October 2023 (KST), were unlikely to be influenced by long-range transport from outside Korea.

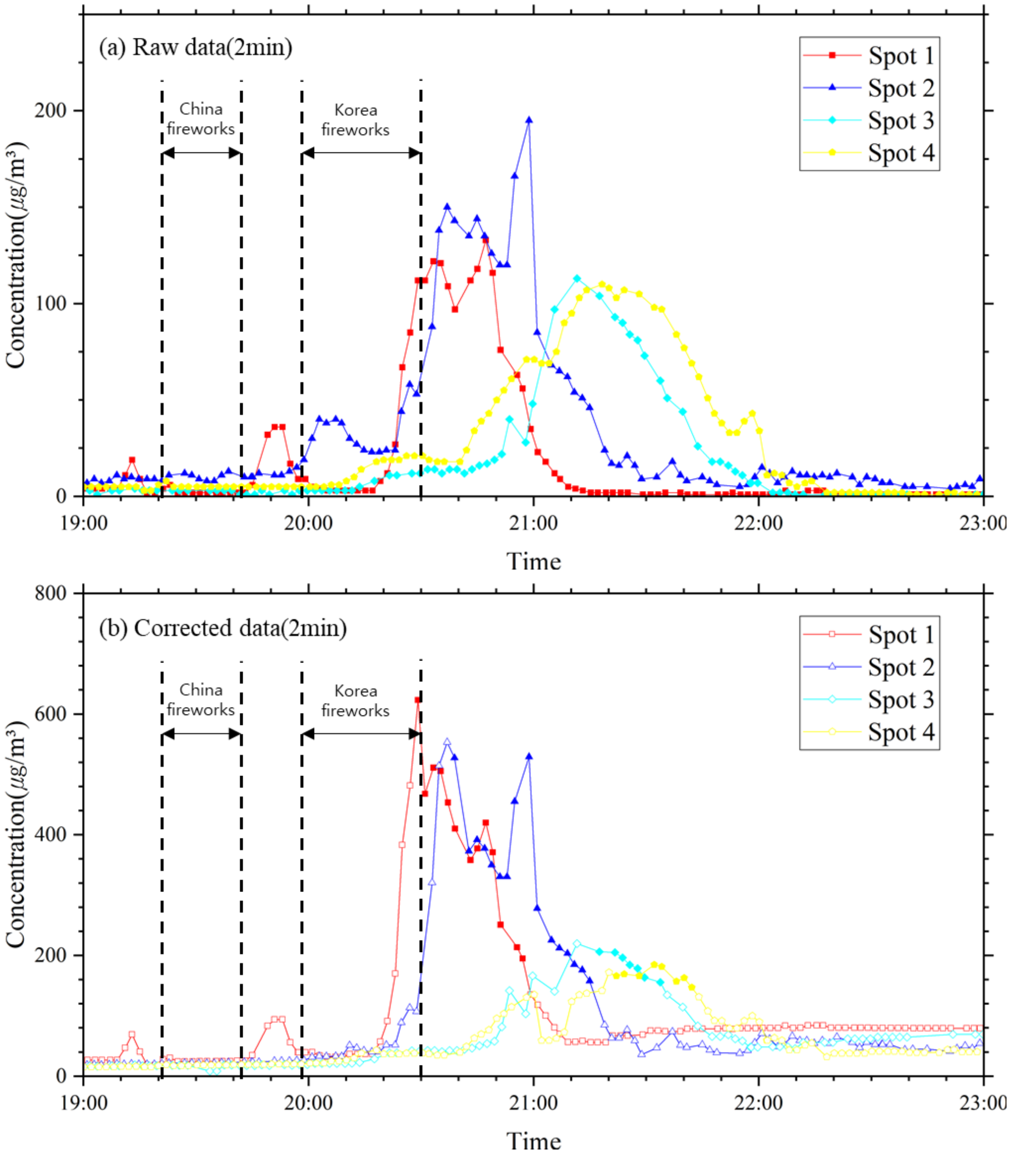

Figure S9. PM_{10} concentrations measured at S-Dot sites during the fireworks festival (19:00–23:00 on 7 October 2023) (a) before and (b) after correction. The vertical dashed lines indicate the duration of the fireworks displays by Team China and Team Korea. In panel (b), filled symbols represent instances when alerts were issued.

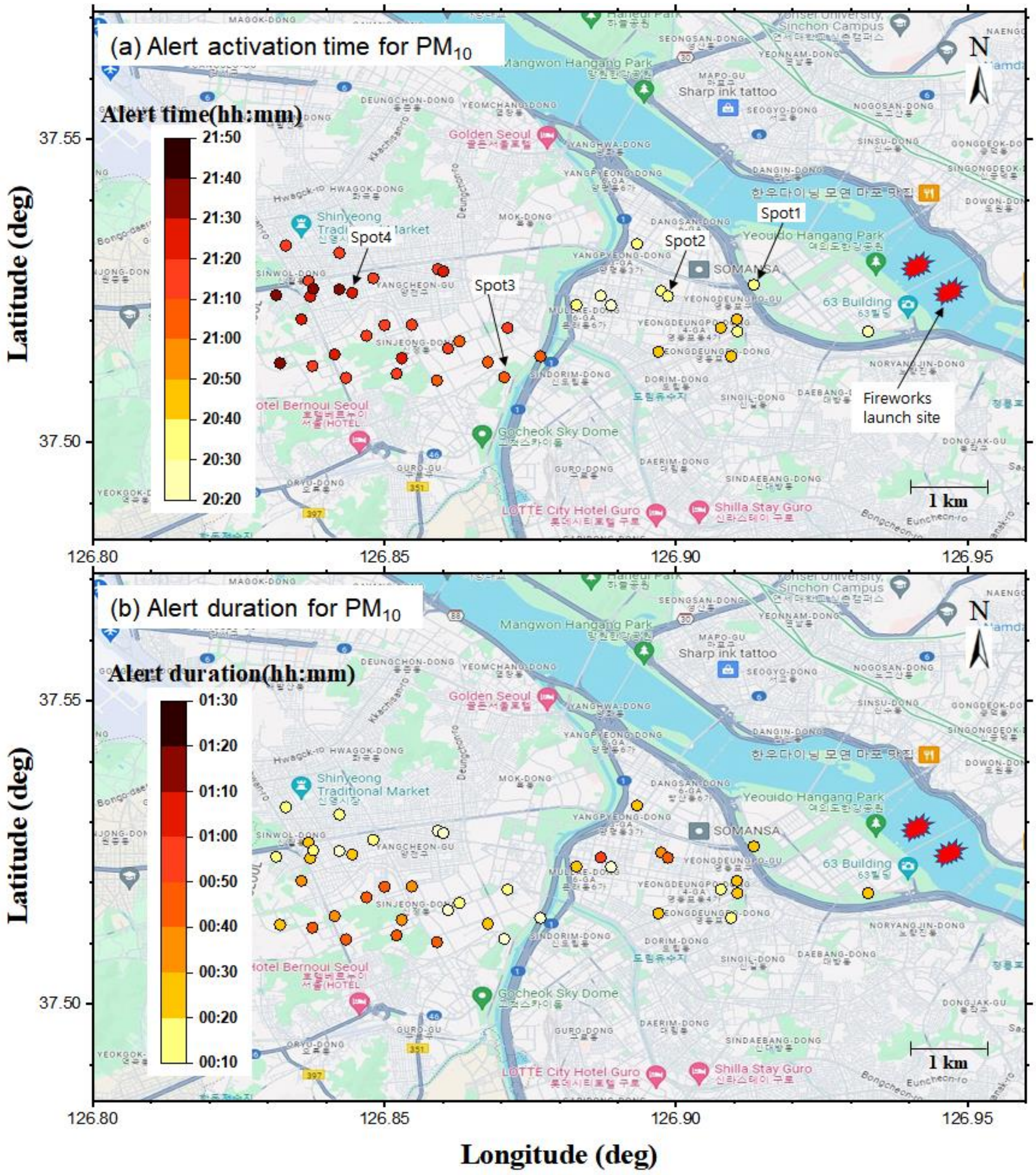

Figure S10. Application results of the new alarm protocol to the fireworks case on 7 October 2023. (a) Alert activation time for PM_{10}; (b) alert duration for PM_{10}. The red burst symbols indicate the fireworks launch sites.
